# Perspective: microbial interventions in the urinary tract

**DOI:** 10.20517/mrr.2022.17

**Published:** 2023-02-17

**Authors:** Gregor Reid

**Affiliations:** ^1^Canadian R&D Centre for Human Microbiome and Probiotics, Lawson Health Research Institute, London, Ontario N6A 4V2, Canada.; ^2^Departments of Microbiology and Immunology, and Surgery, Western University, London, Ontario N6A 3K7, Canada.

**Keywords:** Urinary tract, probiotics, beneficial microbes, infection, calculi

## Abstract

Despite multiple advances in medicine, the management of urinary tract infections (UTIs) in women has remained stalled for decades. To prevent the development of symptomatic recurrences, low-dose antibiotics are the mainstay, while alternative approaches have been attempted with limited success. The use of probiotics was first considered forty years ago, and while some promising studies have been published, additional evidence in larger patient groups is needed to recommend specific strains as a primary preventive regimen. Overall, the role of beneficial microbes in reducing the risk of UTI and other urological diseases, such as urolithiasis, remains a target for researchers. The aim of this perspective is to offer a viewpoint on the status of this approach and recommendations for how to develop novel probiotic therapies.

## INTRODUCTION

It has been almost fifty years since Bruce *et al*. published a study suggesting a potential protective role of lactobacilli in urinary health. That clinical observation provided a rationale for examining lactobacilli properties that might counter uropathogenesis^[1]^. The outcome was the selection of *Lacticaseibacillus* (formerly* Lactobacillus) rhamnosus* GR-1 and *Limosilactobacillus *(formerly* Lactobacillus) reuteri* RC-14 two strains that could inhibit the growth and adherence of the most common UTI causative agents, *Escherichia coli* and *Enterococcus faecalis* through their ability to produce lactic acid, bacteriocin-like compounds and biosurfactants^[2,3]^. Clinical studies in which the strains were administered intravaginally^[4]^ and orally^[5-7]^ showed encouraging results but were not sufficient in terms of sample size and confirmatory evidence to be recommended for prophylaxis. The selection of strains because their species is the most dominant in the vagina is based on a weak foundation^[8]^; although *Lactobacillus crispatus* CTV05 have shown promise as a probiotic to reduce the incidence of UTI^[9]^, it has not been further developed as a product.

In addition to infection, kidney stones remain a major unresolved problem in urology. The vast majority of stones are caused by urinary oxalate^[10]^. For kidney stone formation, the long-held understanding was that crystalline formation is independent of bacteria except for struvite stones where urease-producing strains such as *Proteus*, along with *E. coli* and *Pseudomonas* spp. have been found^[11]^. The considered role of bacteria was only in the gut, where some strains could degrade oxalate. However, the isolation of bacteria from ureteral stents^[12]^ and the ureters and stones of non-stented, asymptomatic patients^[13]^ shows their ability to ascend to the kidney. This raises questions about what they are doing in that nutrient-rich niche and whether they could be involved in non-oxalate stone formation.

The aim of this perspective is to offer a viewpoint on whether beneficial microbes may or may not work to improve urinary health as it pertains to infection and calculi.

## IS THE HYPOTHESIS CORRECT?

Multiple reviews, meta-analyses, opinion pieces and microbiome studies have attempted to uncover mechanisms of acquiring a UTI and different interventions to prevent them^[14,15]^. The evidence clearly points to a long-held view that the pathogens ascend from the rectum to the perineum, vagina and urethra, then into the bladder. This may be further aided by organisms from the vagina entering the bladder and causing damage that makes it easier for uropathogens to infect^[16]^. This reaffirms the concept that interfering with this ascension could be an effective means to prevent recurrence of infection. Indeed, antibiotic prophylaxis is designed to kill pathogens as they ascend.

The probiotic approach postulates that pathogen ascension can be prevented through competition at the perineum and urethra as well as in the intestine where these organisms originate. Studies that aim to increase lactobacilli and decrease pathogens in the urogenital area through probiotic administration, orally or vaginally, show promise in reducing UTI episodes^[4,9,17,18]^, though not all studies report this effect^[19]^. The problem with the vaginal route of administration is that it requires products to be delivered as drugs, thereby increasing the wait time for clinical evidence to be generated. For orally administered approaches, larger study sample sizes are required to get unequivocal evidence of efficacy. Since probiotic strains do not colonize, any therapy will need to be given for a long time.

The vaccine approach would, in theory, prevent uropathogen ascension plus inhibit adherence of the pathogens if they reach the bladder. However, vaccine studies have been mostly in rodents, and so far, none have borne fruit. One group with perhaps the most advanced design of a vaccine has noted that pre-existing antibodies to uropathogenic *E. coli* already exist in adults, even those without a history of UTI^[20]^. This suggests past exposure to the organism, and the need for any vaccine to boost the immune response beyond these baseline levels. Whether or not that is possible remains to be determined. A newer and potentially exciting development of innate immunomodulation therapy targets “bad inflammation” by correcting specific innate immune defects such as IL-1β, MMP7, COX2, cAMP and the pain-sensing receptor NK1R^[21]^. Though mostly tested in animals, an off-label IL-1 receptor antagonist has shown encouraging early results in treating severe bladder pain syndrome patients^[22]^.

In reviewing the literature, the same message as we and others conveyed in the 1980s was repeated that new methods are urgently needed to prevent and treat UTI. The implication is that either researchers are not innovative or their efforts are not being funded sufficiently.

A more radical option that has been explored is to administer a fecal microbiota transplant (FMT), presumably to counter the ascension of uropathogens from the rectum as well as potentially re-set some immunological functions that are not protecting the host. Although in its infancy, case reports have shown successful resolution of recurrent UTI^[23]^, however in those in which FMT was given to resolve *Clostridioides difficile* infection, UTI still subsequently occurred^[24]^. If FMT or probiotics are to be developed, which strains hold the key?

## HOW TO SELECT PROBIOTIC STRAINS TO REDUCE THE RISK OF UTI AND CALCULI?

Forty years ago, the selection of properties deemed appropriate for a strain to act as a probiotic for urinary tract health were their ability to adhere to surfaces and inhibit the growth and attachment of pathogens. However, our understanding of the disease process and properties of lactobacilli and bifidobacteria have progressed and different selection criteria should be considered.

The genome sequence of a strain will identify its potential to produce bacteriocins and use different nutrients for growth. In addition, if it contains antibiotic resistance genes that could possibly transfer to pathogenic organisms, such as *Enterococcus faecium* with vancomycin resistance genes horizontally transferring to pathogenic *Enterococcus faecalis*^[25]^, either the strain will not be approved or the genes will have to be deleted^[26]^. To date, there are no documented cases of probiotic strains being proven to have passed resistant genes to a pathogen that then infected the host and was not treatable. Indeed, an argument has been made that probiotics may actually reduce the spread of antibiotic resistance^[27]^.

The metabolic output of candidate probiotic strains under different conditions determines their ability to produce short-chain fatty acids, biosurfactants, acids and hydrogen peroxide. The ability to up-regulate epithelial junction proteins or antimicrobial compounds could be added as a test for potential application^[28]^. A new concept suggests that some lactobacilli have the ability to dampen ATP production and impede Ca^2+^ influx and contraction, which could be an assessment tool^[29]^, as could the ability of certain *Bifidobacterium* strains to internalize the renal toxin *p*-cresol^[30]^. The latter experiments were done in a *Drosophila* model, which is a useful tool for assessing parameters of urolithiasis^[31]^.

The difficult part becomes weighing the findings to select candidate strains. For example, do you give more importance to activity against *E. coli* than *Enterococcus*? How do you gauge the level of growth inhibition *in vitro* versus what would be required *in vivo*? If there are drug-resistant genes on a plasmid, will regulators insist that the plasmid be removed? Is the ability to coaggregate with pathogens or disrupt biofilm formation important enough to add to the tests? What if a candidate strain is challenging to grow, and therefore if selected, it may not be scaled up to product specifications? This potentially could be the case with some *Lactobacillus iners* strains^[32]^.

The combining of strains GR-1 and RC-14 to inhibit Gram positive and Gram negative uropathogens was the predecessor to a growing number of probiotic products containing multiple strains. Should we know the dynamics of these collectively, how they interact with each other and if the consortia are essential to outcompete the pathogens and restore homogeneity? Or select strains to allow for variability in the recipient hosts, with one probiotic strain being beneficial in some women, another being more receptive in other women?^[33]^

There have been no reports to suggest that hosts have different receptivity to different lactobacilli; however, it would be interesting to investigate whether gene conversion of carcinoembryonic antigen-related cell adhesion molecules (CEACAMs) on urogenital cell surfaces might play a role in which strains can colonize^[34]^.

The ultimate series of experiments should be performed with strains suitable for the target activity. All too often, this is not done. Instead, studies use whatever strains are commercially available, rather than there being proof of desired properties for the urinary tract.

## HOW TO DELIVER THE STRAIN(S)?

There are many factors to consider when deciding the formulation. These include the ten shown in Table 1.

**Table 1:** Factors to consider when deciding on the formulation of a probiotic for application to improve urinary tract health

	**Factors to consider when deciding on the formulation**
(i)	If the strains require protection from stomach acid and bile
(ii)	Any differences in stability between strains
(iii)	The concentration of organisms, therefore, the volume of the dose
(iv)	If a prebiotic or other substances like multivitamins are to be included
(v)	The properties of the vehicle for allergic, religious and other considerations, for example, respecting vegan, kosher and halal requirements
(vi)	The storage conditions and how these affect the viability
(vii)	Whether it is to be a food, supplement, medical device or regulated as a drug
(viii)	How it will be delivered, namely orally or locally to the skin or urogenital orifice
(ix)	The environment (pH, types of mucins, aerobic/anaerobic, osmolality, presence of other organisms and immune defenses) into which the strains will be exposed, as this differs in the small and large intestine, perineum and vagina
(x)	Ease and compliance of administration to account for consumers/patients dexterity and the ability to swallow, as well as presumed preference in the type of product the population wants

Each of the above has ramifications for the process of taking research data and making an end product suitable for human use. These include the assessment of strain properties as well as whether the product will function better with the addition of prebiotics or multivitamins. Consideration must be given to the population that is being targeted and the form in which the product is preferred. Identifying the speed at which commercialization is to take place will influence the process, as the drug regulatory route will take significantly longer. This affects the company that is taking the product to market with implications for the type of delivery method (food, supplement, pharmaceutical, medical device) as well as where consumers/patients go to purchase them (pharmacy, health food store, grocery). This also influences the media outlets that the company will use to market the product, as these can range from print to television, web-based, social media, direct marketing and face-to-face events.

The actual target and desired effect are obviously important, but without the right company and formulation, it may be impossible to deliver. If the intent is to reduce the recurrence of UTI, then scientifically, what would be the optimal way of doing this? Based on the discussion above, direct application would be the primary option for allowing the strains to interact with pathogens in the perineum and urethra in a way that enhances host immunity and creates an environment conducive to the return of the woman’s own beneficial microbiota. Depending on the country and the claims being made, this will fall into the category of medical devices, cosmetics or drugs, each with its own set of required regulations.

With this decision made, the next step is to choose a formulation, presumably a cream/ointment for local application or a capsule/tablet for vaginal insertion. These present their own challenges, but there have been studies on lactobacilli in creams^[35,36]^ and two products have been approved for intravaginal instillation in Canada^[37-39]^. One of the strains, *Lacticaseibacillus rhamnosus* Lcr35, was chosen because of properties believed to be suitable for vaginal application, namely anti-*Candida* activity described in a series of experimental studies^[40]^. In neither of these approved products were prebiotics, supplements or substances of potential benefit to the urogenital tract included in the formulation. An advantage of a multi-strain or multi-product approach for prevention could be, for example, to include a *Bifidobacterium* strain to enhance folate^[41]^ or add estriol for products targeting post-menopausal women^[42]^. This further raises the question of whether products should contain multiple species and strains to restore and maintain homeostasis and reduce the risk of recurrences, or if such a microbiome could be instilled into the bladder to actually treat recalcitrant infections or calculi.

In aiming for an effect in the kidneys, the oral delivery of probiotic bacteria would have to result in the production of substances that reach the kidneys or block toxins from causing damage there. Or their aim would be to degrade oxalate in the gut and reduce the amounts reaching the kidneys. The initial promise that *Oxalobacter formigenes* would be an effective probiotic has not borne fruit in clinical studies, possibly because it requires oxalate intake. Lactobacilli are called “generalist oxalotrophs” since, in contrast to *O. formigenes*, they can grow with and without requiring oxalate^[43]^. Not many strains of *Lactobacillus* and *Bifidobacterium* have oxalyl coenzyme A decarboxylase gene (*oxc*) involved in oxalate degradation^[43]^, but *L. acidophilus* NCFM has an operon with genes homologous to formyl coenzyme A transferase gene (*frc*) and *oxc*, and it is already used as a probiotic taken orally^[44]^.

Until microbiome studies proved otherwise, the thought of bacteria being in a healthy kidney seemed ridiculous. Likewise, instilling a probiotic strain into the organ is not yet being viewed as a realistic option, though that might change in the future. One problem would be how to insert a strain such as NCFM into the kidney microbiome and have it persist and express oxalate-degrading genes without inducing infection or inflammation. Ignoring the difficult ethical issues, a ureteral stent with NCFM at its tip may provide a delivery system for this purpose in patients where other management methods have failed. In fact, the concept of coating ureteral stents with oxalate-degrading enzymes was advanced twenty years ago^[45]^, yet still awaits clinical testing. 

The ultimate test for strain delivery will come from human studies. These can begin with small sample sizes to get a better idea of safety, ease of delivery, patient compliance and potential for the desired primary outcome to be achieved. In my view, this is much more informative than animal studies, though clearly, it requires ethical approval which in some institutions may not be forthcoming unless an investigational new drug document has been prepared.

## CONCLUSIONS

It seems unconscionable that half a century since there was a declared urgent need for major advances in the management of UTI in women, little progress has been made in prevention and treatment. New drugs are being developed for situations when patients have life-threatening carbapenem-resistant Gram-negative infections^[46]^. But alternative preventive therapies remain extremely limited. Fortunately for patients with urolithiasis, the introduction of extracorporeal shock wave lithotripsy has at least provided an effective treatment for many cases. In both diseases, the use of beneficial microbes continues to be investigated as an alternative remedy. To build upon encouraging findings to date, careful identification and documentation of probiotic strain candidates or FMT compositions must be done using modern scientific approaches. In addition, when choosing the delivery system, a myriad of factors that are critical to success must be taken into account.

## DECLARATIONS

### Copyright

© The Author(s) 2023.
